# Zinc/Catechol Resin-Based Microsphere Coating for Durable Antibacterial Cotton Fabrics

**DOI:** 10.3390/polym18101266

**Published:** 2026-05-21

**Authors:** Jun-Xiang Xiong, Zi-Han Yin, Lian-Yi Qu, Ying-Jun Xu

**Affiliations:** Institute of Functional Textiles and Advanced Materials, Shandong Key Laboratory of Polymeric Materials Recycling and Upcycling, National Engineering Research Center for Advanced Fire-Safety Materials D&A (Shandong), College of Textiles & Clothing, Qingdao University, Qingdao 266071, China

**Keywords:** zinc oxide nanoparticles, antibacterial cotton fabric, catechol–formaldehyde resin, adhesion

## Abstract

Zinc oxide nanoparticles (ZnO NPs) exhibit strong and broad-spectrum antibacterial properties, making them a promising agent for textile applications. However, their weak adhesion to fibers and poor washing durability have hindered practical use. In this work, we report zinc/catechol resin-based microspheres (Zn/CFRs) synthesized via a one-pot hydrothermal route and applied to cotton fabric through a pad-dry-cure process. The resulting Zn/CFRs exhibit a monodisperse spherical morphology, with zinc ions concentrated on the surface and ZnO NPs encapsulated within the resin matrix. The finished fabric demonstrates potent, non-leaching antibacterial activity, achieving over 99.99% inhibition against *S. aureus*, *E. coli*, and *C. albicans*, with excellent performance retention even after 50 laundering cycles. Furthermore, we observed that catechol oxidation in the Zn/CFRs proceeds slowly under UV light, which may contribute to the durable adhesion of the coating. Moreover, the functional finishing does not compromise the fabric’s tensile strength, hand feel, or breathability, which positions it favorably for scalable adoption in functional textile manufacturing.

## 1. Introduction

The uncontrolled proliferation and rapid transmission of microorganisms pose a persistent threat to public health [1,2,3]. Textiles, especially cotton fabrics, are widely used and can readily act as carriers for microbial adhesion and proliferation due to their moisture absorption and breathability [4,5]. Therefore, the rising market demand for antibacterial cotton fabrics is driving the global industry’s continuous expansion [6,7]. Current industrial production primarily relies on post-finishing processes, where antimicrobial agents are applied to the surface of finished fabrics to achieve scalable manufacturing [8,9]. Commonly employed agents include metallic nanomaterials, chitosan, *N*-halamines and quaternary ammonium compounds [10,11,12,13,14,15,16]. Among these, metallic nanomaterials have become the most prevalent choice for large-scale production due to their high antibacterial efficiency and relatively straightforward application processes, with silver nanoparticles (Ag NPs) being a representative system [17,18,19]. However, the potential release of Ag^+^ ions raises concerns regarding environmental accumulation and long-term safety [20,21,22]. Consequently, research attention has shifted toward other metal oxide systems with greater potential for environmental friendliness.

Zinc oxide nanoparticles (ZnO NPs) have emerged as a promising alternative, owing to their broad-spectrum efficacy, milder ion release profile, and improved biocompatibility [23,24,25]. However, their weak adhesion to fibers leads to poor durability. To address this, resin binders such as polyurethane or polyacrylate are commonly introduced during pad-dry-cure processes [26,27]. While effective in improving durability, these binders tend to form continuous films on the fabric surface, blocking fiber interstices and thereby deteriorating flexibility and air permeability.

A potential strategy is to construct pre-assembled ZnO-binder composites at the micro/nano scale, which can act as discrete anchoring sites during fabric finishing, avoiding the formation of continuous films. Catechol–formaldehyde resin (CFR), a mussel-inspired adhesive material, is a promising binder due to its strong adhesion to diverse substrates via covalent bonding, hydrogen bonding, and metal coordination. Moreover, CFR exhibits notable advantages, including good water dispersibility, low cost, simple preparation, and scalability. These properties have led to its widespread recent use in nanoparticle surface modification and the fabrication of functional micro/nanostructures [28,29]. Oxidizing ions like Ag^+^ and Fe^3+^ readily undergo redox reactions with CFR, resulting in the in situ generation of Ag nanoparticles or Fe_3_O_4_ nanoparticles. In earlier studies, we utilized this mechanism to prepare Ag/CFR composite microspheres and securely modified them onto cotton fabric through a pad-dry-cure method, producing durable antibacterial textiles with good wearability [30]. Yet, for non-oxidizing ions such as Zn^2+^, zinc-containing CFR systems have been far less studied, and their extension to antibacterial textiles has rarely been explored.

In our previous work [31], Zn/CFRs were prepared using catechol, hexamethylenetetramine, and ZnCl_2_ via a one-pot hydrothermal method. Among them, we systematically investigated the parameters, including the molar ratio of catechol to ZnCl_2_, hydrothermal temperature, and reaction time, and successfully prepared a series of Zn/CFRs with diverse morphologies and particle sizes. In the present work, Zn/CFRs synthesized under optimized conditions were applied to cotton fabrics through a pad-dry-cure process to fabricate durable antibacterial textiles. We focus on the interfacial adhesion behavior between the microspheres and cotton fibers, the washing durability of the antibacterial activity, and the photo-driven redox mechanism that contributes to long-term attachment. In addition, the effects of the finishing on fabric tensile strength, hand feel, and air permeability are evaluated.

## 2. Experimental Methods

### 2.1. Materials

Catechol (AR, 99%) was purchased from Tianjin Damao Chemical Reagent Co., Ltd., (Tianjin, China); HMT (AR, 99.5%) was provided by the Kelong Chemical Corporation (Chengdu, China); and ZnCl_2_ (AR, 99.8%) was supplied by Sinopharm Chemical Reagent Co. Ltd. (Shanghai, China). Cotton fabrics (COT) were acquired from Qirong Textile Printing and Dyeing Co., Ltd. (Weifang, China). The fabrics were cleaned by ultrasonic washing in deionized water before use. Standard strains of *Staphylococcus aureus* (*S. aureus*, CMCC(B)26003), *Escherichia coli* (*E. coli*, CMCC(B)44102), and *Candida albicans* (*C. albicans*, ATCC10231) were obtained from Beijing Microbiological Culture Collection Center (Beijing, China).

### 2.2. Preparation of Zn/CFRs and CFR

In our previous work [31], we systematically investigated and optimized the synthesis conditions of Zn/CFR microspheres, and identified the optimal parameters as follows: First, catechol (0.330 g, 3 mmol), HMT (0.210 g, 1.5 mmol), and ZnCl_2_ (0.041 g, 0.3 mmol) were dissolved in deionized water (400 mL). The mixture was vigorously stirred at room temperature for 30 min to obtain a uniform, transparent solution. The solution was then transferred to a 500 mL Teflon-lined stainless-steel autoclave and heated at 160 °C for 6 h for the hydrothermal reaction. After cooling to room temperature, the product was collected and washed with a deionized water/ethanol mixture at 40 °C for 30 min, and then dried at 60 °C. The final product was a dark-brown powder, denoted as Zn/CFRs. For comparison, pure catechol–formaldehyde resin was prepared under the same conditions without adding ZnCl_2_, and the product was denoted as CFR.

### 2.3. Fabrication of Zn/CFR-Coated Fabrics

Zn/CFR microspheres were ultrasonically dispersed in deionized water to prepare aqueous dispersions at concentrations of 1%, 3%, and 5%. Cotton fabrics were cut to size, immersed in the dispersions, and then processed by a two-dip-two-nip padding procedure with 100% wet pickup. The padded fabrics were oven-cured at 100 °C for 10 min, followed by two washes in warm water (45 °C, 45 min each) to remove loosely attached microspheres, and then dried. The resulting durable antibacterial fabrics treated with 1%, 3%, and 5% dispersions were denoted as COT-1, COT-2, and COT-3, respectively.

### 2.4. Characterization

Scanning electron microscopy (SEM) was conducted using a scanning electron microscope (TESCAN, Brno, Czech Republic). The samples were sputter-coated with a thin gold layer using a vacuum sputter coater for 180 s to enhance surface conductivity. Transmission electron microscopy (TEM) was performed using a transmission electron microscope (Hitachi HT7700, Tokyo, Japan). High-resolution SEM and spot-scanning element measurements were made using a field-emission SEM (FESEM) (TESCAN MIRA LMS, Brno, Czech Republic) equipped with an energy dispersive X-ray spectrometer (EDX) (Oxford Instruments, Abingdon, UK). The samples were sputter-coated with gold to improve surface conductivity. X-ray photoelectron spectra (XPS) were recorded on an ESCALAB 250Xi apparatus (ThermoFisher Scientific, Waltham, MA, USA) utilizing Al Kα excitation radiation (hν = 1486.6 eV). CIE LAB color space values of the fabrics were determined using a Datacolor 850 color matching system (Datacolor, Shanghai, China).

Zone of inhibition (ZOI) tests were conducted according to GB/T 31713-2015 [32] to determine whether the antibacterial fabrics were of the dissolution/non-dissolution type, and the antimicrobial activity of the fabrics was then evaluated using a shake-flask method following GB/T 20944.3-2008 [33]. Briefly, a fabric sample was immersed in a microbial suspension and shaken for a defined period, after which the suspension was serially diluted, plated on agar, and incubated at 37 °C for 24 h, and microbial viability was quantified by the plate-counting method to determine antibacterial activity based on colony-forming units (CFUs), with *S. aureus*, *E. coli*, and *C. albicans* used to assess activity against Gram-positive bacteria, Gram-negative bacteria, and fungi, respectively, with each test performed in triplicate. *E. coli* and *S. aureus* were also used as representative strains to evaluate the UV–photocatalytic antibacterial performance of the Zn/CFRs: 100 μL of a bacterial suspension with an initial concentration of 10^5^ CFU mL^−1^ was added to 5 mL of an aqueous dispersion (512 μg mL^−1^) of CFR or Zn/CFRs, the mixture was exposed to 365 nm UV light (+Light) for 30 min or incubated in the dark (−Light) for 30 min, and the samples were irradiated from above at a fixed distance of 20 cm between the lamp and the sample surface. After treatment, the mixtures were transferred onto agar plates and incubated at 37 °C for 24 h, and colony growth was observed to assess the antibacterial effect, with PBS buffer tested in the same way as the control group.

The laundering durability of the treated fabrics was investigated according to AATCC Test Method 61-2013 [34], Test No. 1A, using an SW-20B laundering machine (Quanzhou Meibang Instrument Co., Ltd., Quanzhou, China). Briefly, the treated fabrics were washed in a stainless-steel container at 40 °C with 200 mL of 0.37% (*w*/*v*) WOB detergent solution and 10 steel balls. One standard wash (45 min) was considered equivalent to five household or commercial laundering cycles. After washing, the samples were rinsed several times with distilled water and dried at 60 °C for 0.5 h. The Zn content of the coated fabrics after 10, 30, and 50 washing cycles was measured by inductively coupled plasma–optical emission spectrometry (ICP-OES) using an Avio 200 instrument (PerkinElmer, Waltham, MA, USA).

The evolution of phenolic -OH group intensity in Zn/CFRs and CFR was monitored by in situ Fourier transform infrared (FTIR) spectroscopy. Spectra were acquired in transmission mode on a Nicolet iS50 FTIR spectrometer (Thermo Fisher Scientific, Waltham, MA, USA) using KBr pellets prepared by homogeneously mixing the sample with KBr and pressing. Under continuous UV irradiation, spectra were collected at 5 min intervals over the wavenumber range of 500–4000 cm^−1^, with 32 co-added scans per spectrum.

Tensile testing of the fabrics was performed using a YG065H electronic strength tester (Laizhou Electron Instrument Co., Ltd., Yantai, China) according to GB/T 3923.1-2013 [35]. Hand feel of the fabrics was evaluated using a PhabrOmeter system (Nu Cybertek, Inc., Davis, CA, USA) following AATCC TM 202. Air permeability was measured with a YG461E-III instrument (Ningbo Textile Instrument Factory, Ningbo, China) according to GB/T 5453-1997 [36].

## 3. Results and Discussion

### 3.1. Preparation and Characterization of Zn/CFRs

**Figure 1:**
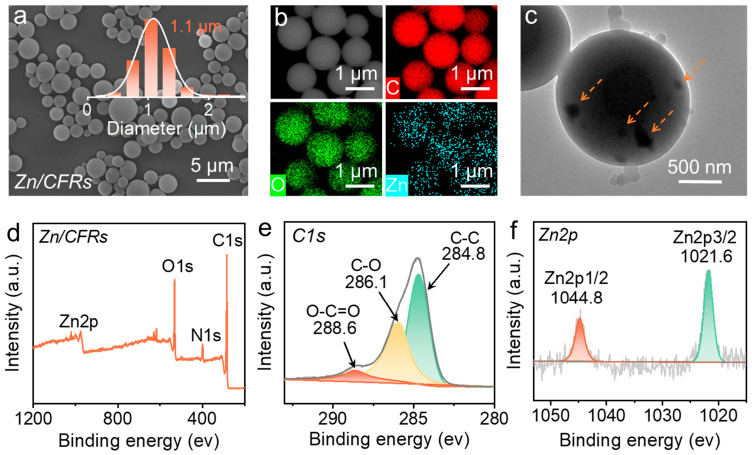
Morphological and chemical structure analysis of Zn/CFRs: SEM images and particle size distributions (**a**), elemental mapping images (**b**), TEM image (**c**), full XPS survey spectrum of the Zn/CFRs (**d**), high-resolution spectra of C 1s (**e**) and Zn 2p (**f**).

The morphology and composition of the Zn/CFRs were systematically characterized. As shown in the scanning electron microscopy (SEM) images (Figure 1a), the Zn/CFRs appeared as uniform, monodisperse microspheres with smooth surfaces. Particle size analysis indicated an average diameter of approximately 1.1 μm. Elemental mapping performed by SEM-EDX (Figure 1b) revealed the presence of C, O, and Zn on the microsphere surfaces, with corresponding weight percentages of 73.2 wt%, 18.3 wt%, and 1.3 wt%, respectively. Notably, while C and O were homogeneously distributed, Zn exhibited a distinct, relatively uniform signal across the surface, suggesting a widespread presence of zinc species on the microsphere exterior. Further structural insight was provided by TEM analysis (Figure 1c), which revealed that irregular nanoparticles were encapsulated within the microsphere matrix, with an average particle size of approximately 50–80 nm. The chemical states of surface elements were investigated by X-ray photoelectron spectra (XPS). The survey spectrum presented in Figure 1d confirmed the presence of C, O, N, and Zn, with the N signal likely originating from NH_4_^+^ byproducts. The high-resolution C 1s spectrum (Figure 1e) exhibited three peaks at 284.8 eV, 286.1 eV, and 288.6 eV, corresponding to C-C, C-O, and O-C=O bonds, respectively. This profile matches the characteristic bonding states reported for catechol–formaldehyde resins, confirming the successful formation of the CFR framework [37,38]. Furthermore, the Zn 2p spectrum (Figure 1f) displayed a characteristic doublet with peaks at 1021.6 eV (Zn 2p_3/2_) and 1044.8 eV (Zn 2p_1/2_), accompanied by a spin–orbit splitting of 23.2 eV [39,40]. This signature is consistent with zinc in an oxidized state (Zn^2+^), confirming the successful incorporation of zinc oxide or similar oxidized zinc species into the composite microspheres. Collectively, these results demonstrated that the Zn/CFRs possessed a well-defined composite architecture where zinc ions were concentrated on the surface and ZnO NPs were encapsulated within the resin matrix.

### 3.2. Characterization and Antimicrobial Properties of Zn/CFR-Coated Cotton Fabrics

**Figure 2:**
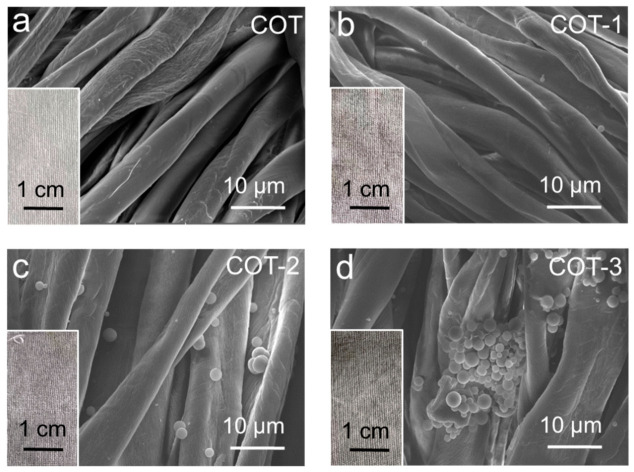
Digital photographs and SEM images of untreated and Zn/CFR-coated cotton fabrics: COT (**a**), COT-1 (**b**), COT-2 (**c**), and COT-3 (**d**).

Figure 2 displayed digital photographs and corresponding SEM images of the cotton fabric samples, including the pristine cotton fabric (COT) as well as the finished fabrics COT-1, COT-2 and COT-3. The COT fabric (Figure 2a) appeared grayish-white, with wrinkled but otherwise smooth and clean fiber surfaces. After treatment with Zn/CFRs, the fabric color darkened progressively with increasing microsphere concentration. COT-1 (Figure 2b) showed a light gray hue and only a sparse deposition of microspheres, mainly within the gaps between fibers. COT-2 (Figure 2c) exhibited a darker gray color and a significantly higher density of microspheres, which covered fiber surfaces, grooves and interstitial spaces. COT-3 (Figure 2d) appeared grayish-black, with microspheres densely enriched on the fabric. At this high concentration, some microspheres formed film-like structures on the fibers, while others aggregated into larger clusters. These results confirmed the successful construction of Zn/CFRs on the fabric via the pad-dry-cure process. Increasing the Zn/CFRs concentration led to a gradual darkening of the fabric color from grayish white to grayish black, alongside a morphological transition from discrete microsphere attachment to pronounced surface aggregation and regional film formation.

**Figure 3:**
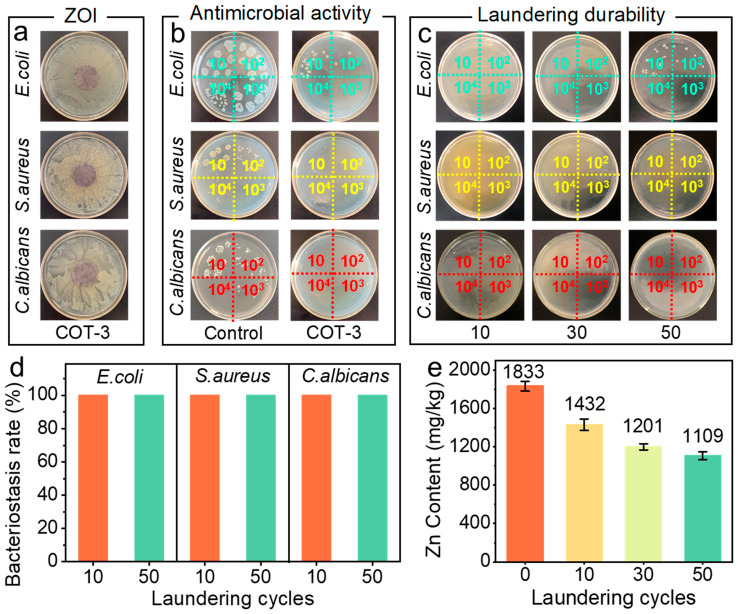
ZOI photographs of COT-3 against *E. coli*, *S. aureus*, and *C. albicans* (**a**). Digital photos of agar plates with microbial suspensions treated with cotton samples before and after laundering cycles (**b**,**c**). Bacteriostasis rate of COT-3 versus the number of laundering cycles (**d**). Zn content of COT-3 versus the number of laundering cycles (**e**).

Using the COT-3 fabric as an example, the dissolution/non-dissolution type of antibacterial activity of the COT-3 fabric was first investigated via the ZOI test. As shown in Figure 3a, no clear inhibition zone was observed around the COT-3 sample against *E. coli*, *S. aureus*, or *C. albicans*, while bacterial/fungal lawns grew densely on the surrounding agar. According to the Chinese national standard GB/T 31713-2015, which specifies the hygienic requirements for antibacterial textiles, COT-3 meets the classification criteria for the non-leaching type. Given this characteristic, the antibacterial performance of COT-1, COT-2, and COT-3 was quantitatively evaluated against *E. coli* using the shake-flask method. Photographs of the agar plates (Figure S1) showed abundant colonies in the 10^2^ dilution areas for the control COT, COT-1, and COT-2. In contrast, almost no colonies were visible for COT-3, demonstrating its high antibacterial efficacy, with the corresponding antibacterial activities of COT-1, COT-2, and COT-3 against *E. coli* summarized in Table S1. Consistently, COT-3 exhibited equally strong activity against *S. aureus* and *C. albicans*, with a bacteriostatic rate exceeding 99.99% (Figure 3b). The washing durability of the fabric was assessed through accelerated laundering tests (AATCC standard), where one laundering cycle represents five equivalent home washes. Remarkably, even after 10, 30, and 50 cycles, the agar plates inoculated with washed COT-3 fabrics showed no or negligible microbial growth (Figure 3c). The corresponding bacteriostasis rates against *E. coli*, *S. aureus*, and *C. albicans* remained as high as 99.99% after 50 washing cycles (Figure 3d), and the results at different washing cycles are summarized in Table S2. To further investigate the morphological evolution after laundering, we performed SEM observations on the COT-3 fabric after 50 accelerated laundering cycles. As shown in Figure S2, a substantial number of Zn/CFRs remained attached to the cotton fiber surfaces even after 50 washing cycles. In addition, the zinc content on COT-3 before and after washing was measured by ICP-OES. As plotted in Figure 3e, the initial Zn content (1833 mg kg^−1^) remained substantial after laundering, retaining 1432, 1201, and 1109 mg kg^−1^ after 10, 30, and 50 cycles, respectively. This high zinc retention can be attributed to the catechol groups in the CFR matrix, which form multivalent hydrogen bonding interactions with the cellulose substrate, ensuring strong interfacial adhesion between the microspheres and the cotton fibers. This robust adhesion contributes to the stable performance of the fabric over repeated washing cycles. Furthermore, our previous work has demonstrated the excellent cytocompatibility of Zn/CFRs toward mammalian cells. Taken together, these properties meet the practical requirements for applications in clothing, bedding, and decorative textiles.

**Figure 4:**
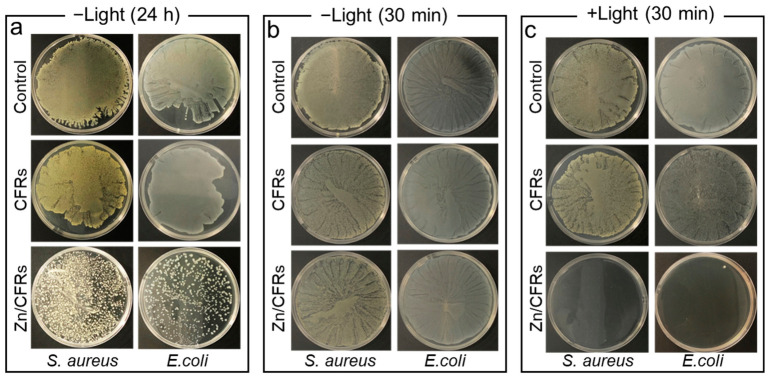
Photos of the agar culture medium corresponding to the co-cultivation of Zn/CFRs, CFR and PBS with *E. coli* and *S. aureus*: under dark (−Light) conditions for 24 h (**a**), under dark (−Light) conditions for 30 min (**b**) and under ultraviolet light (+Light) conditions for 30 min (**c**).

Zn/CFRs contained Zn^2+^, ZnO NPs, and the CFR matrix as potential antibacterial components. The antibacterial mechanism of Zn/CFRs was investigated using *E. coli* and *S. aureus* as representative model strains. As shown in Figure 4a, abundant bacterial colonies were observed for both the control and CFR-treated groups after 24 h dark incubation, indicating the CFR matrix and catechol moieties do not exhibit obvious antibacterial activity. In contrast, Zn/CFRs led to a pronounced reduction in colony counts for both strains. Considering that ZnO NPs are primarily confined within the microspheres, while Zn^2+^ are distributed both internally and externally, the antibacterial effect under dark conditions is mainly attributed to the release of Zn^2+^, which is also the primary antibacterial mechanism of COT-3.

Given the reported photocatalytic properties of ZnO NPs in the literature, we further evaluated the antibacterial efficacy of the Zn/CFRs under UV light irradiation [41,42]. The experiment involved two treatment conditions: one group was exposed to 365 nm ultraviolet light (+Light) for 30 min, while the other was kept in the dark (−Light) for the same duration. After treatment, the bacterial suspensions were spread onto agar plates, and the antibacterial efficacy was evaluated by observing colony growth. Under dark conditions, substantial bacterial growth was observed on the agar plates corresponding to the control group, the CFR group, and the Zn/CFR group, indicating that the catechol groups, ZnO NPs and Zn^2+^ did not exhibit rapid bactericidal activity within 30 min (Figure 4b). Following UV light irradiation, the plates of the control and CFR groups still showed abundant colony formation, whereas the surface of the Zn/CFR plates displayed nearly no bacterial growth, demonstrating the pronounced photocatalytic antibacterial activity of Zn/CFRs under light exposure (Figure 4c). Figure S3 showed the hydroxyl radical signals of Zn/CFRs under light and dark conditions. Under UV irradiation, a characteristic four-line signal of hydroxyl radicals was observed in the Zn/CFR dispersion, whereas no such signal was detected in the dark. These results indicated that ZnO NPs within the Zn/CFRs could generate ROS in aqueous environments under UV light, which is considered a key factor contributing to their enhanced antibacterial activity. These ROS directly damage bacterial cellular structures, leading to effective bacterial inactivation [43,44]. Unfortunately, the application of this UV-induced antibacterial effect on cotton fabrics is limited, as the fabrics themselves are susceptible to UV degradation. However, the photocatalytic activity of the Zn/CFRs still holds promise for other potential applications.

**Figure 5:**
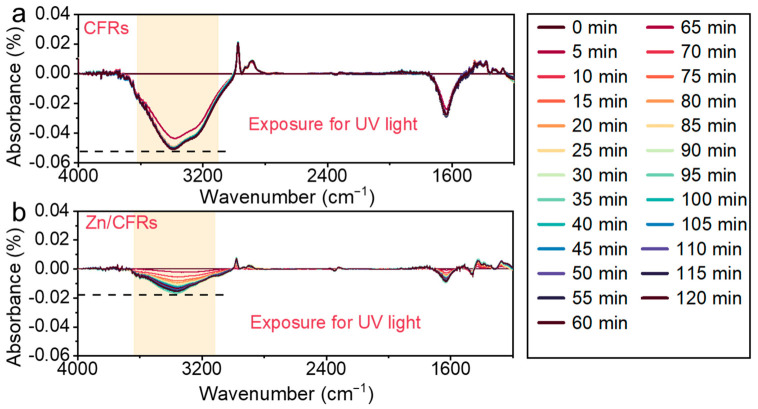
In situ FTIR spectrogram of CFR (**a**) and Zn/CFRs (**b**) under different ultraviolet irradiation times.

Mussel-inspired adhesion has been extensively studied, and it is well established that a dynamic redox equilibrium between catechol and quinone groups plays a key role in durable adhesion, particularly in the presence of metal species such as silver nanoparticles [45,46]. In our previous work on Ag/CFR systems, we observed a similar redox cycling phenomenon [30]. Accordingly, in situ infrared spectroscopy was employed to further examine the changes in chemical groups within Zn/CFRs under UV irradiation, using CFR as a control. As shown in Figure 5a,b, distinct absorption peaks were observed at 3420, 2931, 1636, and 1443 cm^−1^, corresponding to O-H stretching, C-H stretching, and the characteristic vibrations of the aromatic ring, respectively [47,48]. Notably, the intensity of the O-H stretching peak in both CFR and Zn/CFRs changed under UV irradiation. Specifically, the peak intensity decreased rapidly in CFRs but declined much more slowly in Zn/CFRs, indicating oxidation of the -OH groups and a markedly slower oxidation rate in Zn/CFRs. Further analysis focusing on the 80–110 min period of UV exposure revealed that the -OH peak intensity in Zn/CFRs fluctuated rather than decreasing continuously (Figure S4). These phenomena, namely the slower oxidation rate and the observed fluctuations, are interesting and may provide useful insights into the photochemical behavior of catechol–metal systems. Tentatively, this behavior could be linked to the mussel-inspired adhesion concept, potentially contributing to the durable adhesion and sustained antibacterial performance of Zn/CFRs [49,50,51].

### 3.3. Fundamental Properties of Zn/CFR-Coated Cotton Fabrics

**Figure 6:**
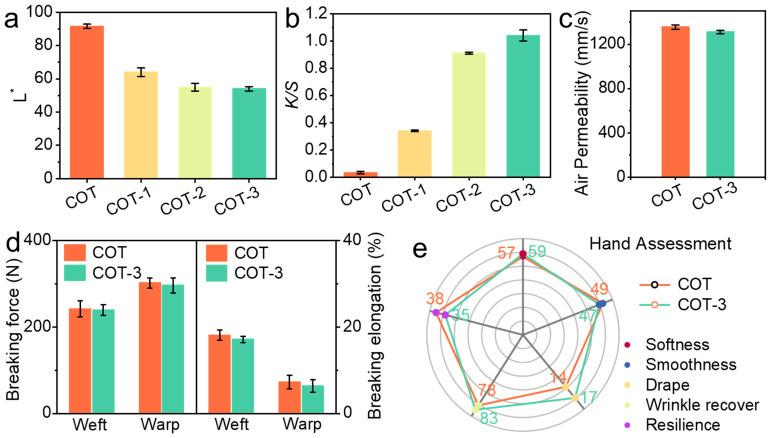
Color parameters of untreated and Zn/CFR-coated cotton fabrics according to the CIE L* (**a**) and *K*/*S* (**b**). Air permeability (**c**), tensile test (**d**) and hand assessment (**e**) of COT and COT-3.

The color, air permeability, mechanical strength, and hand feel of the fabrics were evaluated, as these properties are critical for determining the material’s suitability in practical applications. Color changes were assessed using CIE L*a*b* coordinates and *K*/*S* values. As shown in Figure 6a, the lightness (L* value) decreased from 91 for the control fabric to 64, 55, and 54 for COT-1, COT-2, and COT-3, respectively, indicating a gradual darkening. Correspondingly, the *K*/*S* values (Figure 6b) increased from 0.03 for the control to 0.34, 0.91, and 1.04 for the coated samples, confirming enhanced color depth with higher Zn/CFR loading. Air permeability, crucial for breathability, was tested according to GB/T 5453-1997. As presented in Figure 6c, the coating caused only a minor reduction, from 1355 mm s^−1^ to 1312 mm s^−1^, corresponding to a retention of 96.7%. Compared with previously reported antibacterial cotton textiles, COT-3 exhibited comparable or even superior antibacterial activity and washing durability while exerting minimal impact on air permeability (Table S3) [52,53,54,55,56]. This minimal impact is attributed to the random and regional adhesion of Zn/CFRs on fiber surfaces, without forming a continuous film that would block fabric pores. The tensile properties were nearly unaffected by the coating. Figure 6d shows that both the tensile strength and breaking elongation of COT-3 in the warp and weft directions remained almost identical to those of the uncoated fabric, indicating negligible influence on mechanical integrity under mild processing conditions. Finally, the hand feel was evaluated instrumentally. Key parameters, including softness, resilience, and smoothness, are presented in Figure 6e for comparison. The scores for COT-3 (57, 38, 49) showed minimal deviation from those of the untreated fabric (59, 35, 47), demonstrating that the coating largely preserved the inherent and desirable tactile characteristics of the original cotton.

## 4. Conclusions

In this work, Zn/CFRs were successfully synthesized via a one-pot hydrothermal method using catechol, HMT, and ZnCl_2_ as precursors. The Zn/CFRs exhibited monodisperse microspheres, with zinc ions and ZnO NPs present on the surface and within the microspheres. Thanks to the catechol functional groups in the CFR, the Zn/CFRs had strong adhesion, allowing them to firmly bond to cotton fabric surfaces through a pad-dry-cure process. The coated fabric (COT-3) exhibited non-leaching antibacterial behavior, with 99.99% inhibition against *E. coli*, *S. aureus*, and *C. albicans* maintained even after 50 laundering cycles. Furthermore, under UV light, the Zn/CFRs exhibited both potent photocatalytic antibacterial activity through the action of ZnO NPs and the ability to retard the oxidation of phenolic hydroxyl groups, which may contribute to their durable adhesion. Moreover, the Zn/CFRs and finishing process had no significant effect on the fabric’s tensile strength, hand feel, or breathability. Given the efficient antibacterial activity, durable washing resistance, and light-responsive dynamic adhesion demonstrated by Zn/CFRs, this study presents a practical and scalable strategy for producing long-lasting, high-performance antibacterial textiles, showing promising potential for applications in medical textiles, outdoor functional apparel, and protective gear. However, we note that the contribution of UV-induced phenolic hydroxyl redox behavior to durable adhesion, as well as the UV stability of the Zn/CFR-coated fabric, have not been fully validated, and both aspects will be addressed in future work.

## Data Availability

The raw data supporting the conclusions of this article will be made available by the authors on request.

## Supplementary Materials

The following supporting information can be downloaded at: https://www.mdpi.com/article/10.3390/polym18101266/s1, Figure S1: Curve graph of hydroxyl radicals produced by Zn/CFRs under ultraviolet light (+Light) and in the absence of light (−Light); Figure S2: SEM image of COT-3 after 50 washing cycles; Figure S3: Photos of the agar medium for co-culturing COT, COT-1, COT-2 and COT-3 with *E. coli*; Figure S4: In situ FTIR spectrogram of the -OH stretching vibration peak of Zn/CFRs and CFR under different ultraviolet light irradiation times; Table S1: Antibacterial activity of COT-1, COT-2 and COT-3 against *E. coli*; Table S2: Antibacterial activity of COT-3 against *E. coli*, *S. aureus* and *C. albicans* under different washing times; Table S3: Comparison of the properties of different ZnO-based antibacterial cotton fabrics.
