# Supplementary material for: Zinc/Catechol Resin-Based Microsphere Coating for Durable Antibacterial Cotton Fabrics
Source: Polymers (Basel). 2026 May 21;18(10):1266. doi: 10.3390/polym18101266 (PMC13210553; doi:10.3390/polym18101266)
Supplement: Supplementary file 1 [file polymers-18-01266-s001.zip › polymers-4309127-supplementary.pdf]

Supporting Information

for

Zinc/Catechol Resin-Based Microsphere Coating for Durable Antibacterial Cotton Fabrics

Jun-Xiang Xiong, Zi-Han Yin, Lian-Yi Qu* and Ying-Jun Xu*

Institute of Functional Textiles and Advanced Materials, Shandong Key Laboratory of Polymeric Materials Recycling and Upcycling, National Engineering Research Center for Advanced Fire-Safety Materials D&A (Shandong), College of Textiles & Clothing, Qingdao University, Qingdao 266071, China.

Corresponding to lianyi.qu@qdu.edu.cn (L.-Y. Q.); yingjun.xu@qdu.edu.cn (Y.-J. X.)

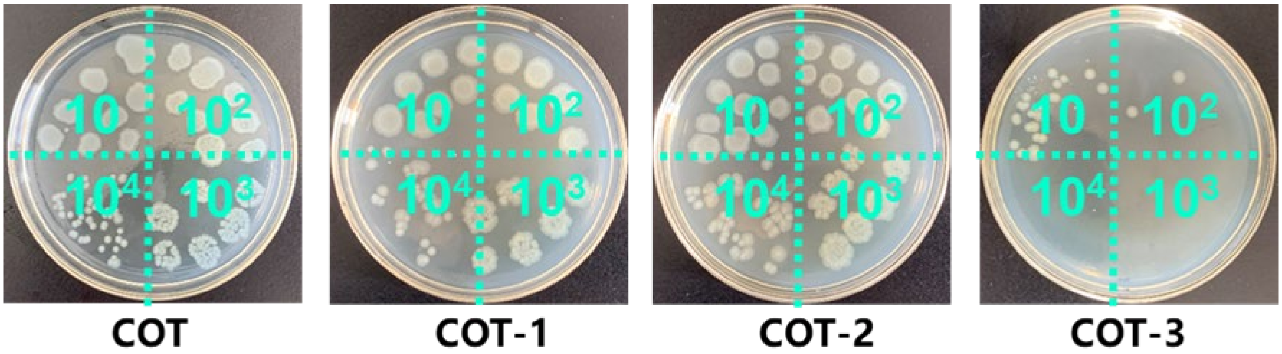

Figure S1 Curve graph of hydroxyl radicals produced by Zn/CFRs under ultraviolet light (+Light) and in the absence of light (-Light).

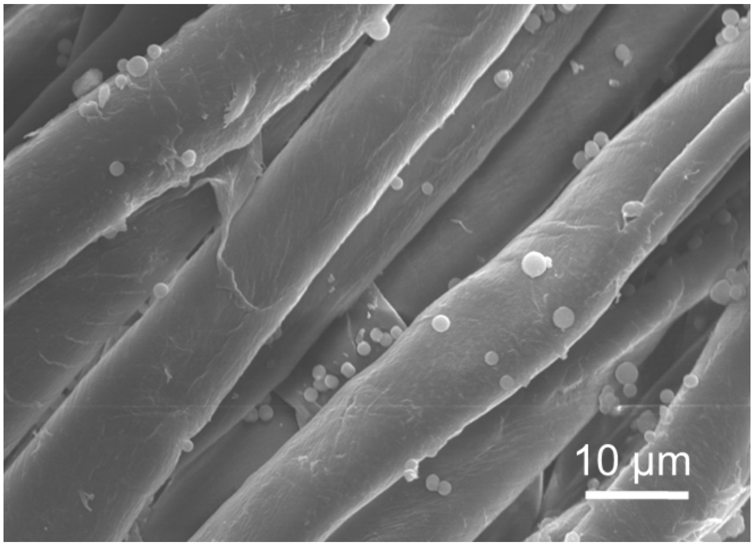

Figure S2 SEM image of COT-3 after 50 washing cycles.

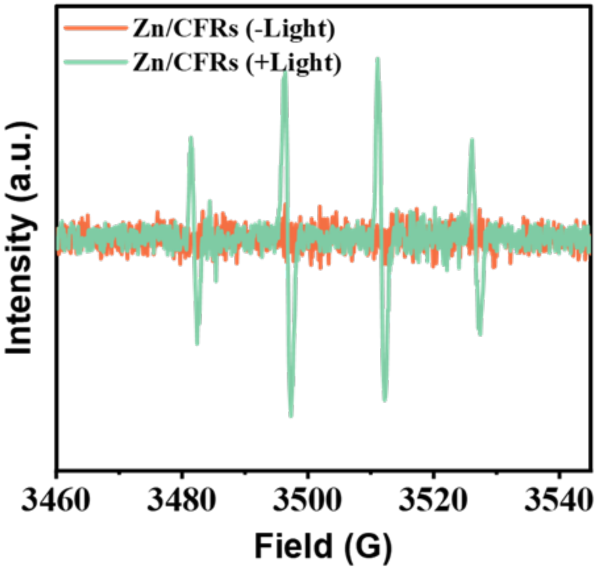

Figure S3 Photos of the agar medium for co-culturing COT, COT-1, COT-2 and COT-3 with *E. coli*.

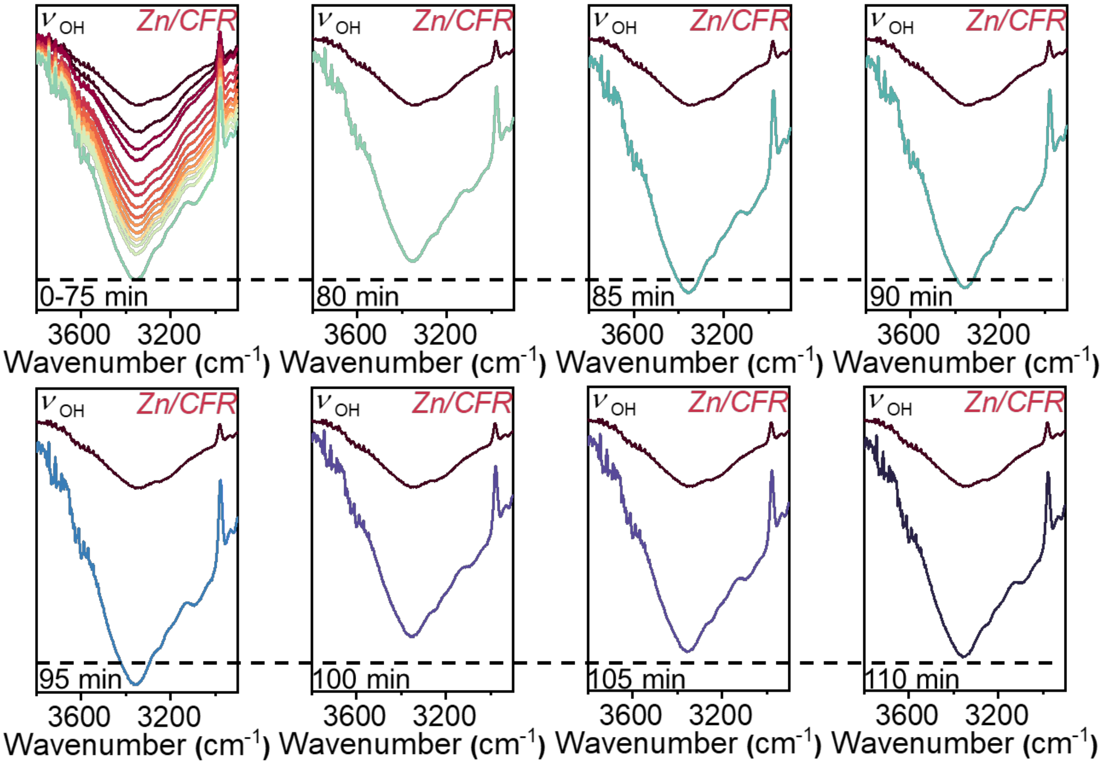

Figure S4 In situ FTIR spectrogram of the -OH stretching vibration peak of Zn/CFRs and CFR under different ultraviolet light irradiation times.

Table S1 Antibacterial activity of COT-1, COT-2 and COT-3 against *E. coli*.

Sample	Antimicrobial Activity (%)
	E.coli
COT-1	11.63
COT-2	30.23
COT-3	99.99

Table S2 Antibacterial activity of COT-3 against *E. coli*, *S. aureus* and *C. albicans* under different washing times.

Sample	Number of Washes	Antimicrobial Activity (%)		
		E.coli	*S. aureus*	*C. albicans*
COT-3	0	99.99	99.99	99.99
	10	99.99	99.99	99.99
	30	99.99	99.99	99.99
	50	99.99	99.99	99.99

Table S3 Comparison of the properties of different ZnO-based antibacterial cotton fabrics.

Antimicrobial Additives	Preparation Method	Number of Washes	Laundering Durability	Air Permeability
ZnO [1]	In situ synthesis	10	93.4%	64.4%
Cu_3P-ZnO [2]	Pad-dry-cure	50	94.7%	93.9%
ZnO [3]	Covalent binding	55	99.0%	69.6%
ZnO [4]	In situ synthesis	30	99.0%	-
BN-Ag [5]	Covalent bonding	20	97.1%	76.3%
**This work**	Pad-dry-cure	**50**	**99.9%**	**96.8%**

References

1. K. Kachare, S.S. Shendage, N. Shinde, S.B. Kashte, S. Vhanbatte, R.-a. Doong, A.V. Ghule, Surfactants increase the durability and antibacterial performance of ZnO-coated cotton, *ACS Appl. Nano Mater.* **2025**, 8, 13563-13572.

2. B. Chen, Y. Huang, Q. Xiao, T. Wang, Y. Hu, X. Chen, F. Chen, W. Lu, Construction of Cu_3P-ZnO coated cotton fabrics with efficient ROS-mediated antibacterial activity, *New J. Chem.* **2026**, 50, 2088-2098.

3. D. Gao, Y. Li, B. Lyu, L. Lyu, S. Chen, J. Ma, Construction of durable antibacterial and anti-mildew cotton fabric based on P(DMDAAC-AGE)/Ag/ZnO composites, *Carbohydr. Polym.* **2019**, 204, 161-

169.

4. Y. Dai, H. Li, J. Wan, L. Liang, J. Yan, Green in-situ synthesis of silver nanoparticles from natural madder dye for the preparation of coloured functional cotton fabric, *Ind. Crops Prod.* **2024**, 208, 117871.

5. S. Bishnoi, G. Aalam, T.A. Mir, S.W. Ali, S.K. Pattanayek, Silver-decorated boron nitride nanocomposite-infused cotton fabric with durable antimicrobial and multifunctional properties, *Nanoscale* **2025**, 17, 23676-23693.
